# WHO Critical Priority Escherichia coli as One Health Challenge for a Post-Pandemic Scenario: Genomic Surveillance and Analysis of Current Trends in Brazil

**DOI:** 10.1128/spectrum.01256-21

**Published:** 2022-03-02

**Authors:** Bruna Fuga, Fábio P. Sellera, Louise Cerdeira, Fernanda Esposito, Brenda Cardoso, Herrison Fontana, Quézia Moura, Adriana Cardenas-Arias, Elder Sano, Rosineide M. Ribas, Albalúcia C. Carvalho, Maria Cristina B. Tognim, Marcia Maria C. de Morais, Ana Judith P. G. Quaresma, Ângela Patrícia Santana, Joice N. Reis, Marcelo Pilonetto, Eliana Carolina Vespero, Raquel R. Bonelli, Aloysio M. F. Cerqueira, Thaís C. M. Sincero, Nilton Lincopan

**Affiliations:** a Department of Microbiology, Instituto de Ciências Biomédicas, University of São Paulo, São Paulo, Brazil; b Department of Clinical Analysis, Faculty of Pharmaceutical Sciences, University of São Paulo, São Paulo, Brazil; c One Health Brazilian Resistance Project (OneBR), São Paulo, Brazil; d Department of Internal Medicine, School of Veterinary Medicine and Animal Science, University of São Paulo, São Paulo, Brazil; e School of Veterinary Medicine, Metropolitan University of Santos, Santos, Brazil; f Department of Infectious Diseases, Central Clinical School, Monash University, Melbourne, Australia; g Department of Vector Biology, Liverpool School of Tropical Medicine, Liverpool, UK; h Faculty of Health Sciences, Federal University of Grande Dourados, Dourados, Mato Grosso do Sul, Brazil; i Laboratory of Molecular Microbiology, Institute of Biomedical Sciences, Federal University of Uberlândia, Uberlândia, Brazil; j Clinical Laboratory, Federal University of Paraiba, João Pessoa, Paraíba, Brazil; k School of Pharmacy, State University of Maringá, Maringá, Brazil; l Institute of Biological Sciences, University of Pernambuco, Recife, Brazil; m Laboratory of Special Pathogen Infections, Bacteriology and Mycology, Evandro Chagas Institute, Ananindeua, Brazil; n Faculty of Agronomy and Veterinary Medicine, University of Brasília, Brasília, Brazil; o Faculty of Pharmacy, Federal University of Bahia, Salvador, Brazil; p Central Laboratory of the State of Paraná-LACEN, São Jose dos Pinhais, Brazil; q Microbiology Laboratory of the University Hospital of Londrina, State University of Londrina, Lonrina, Brazil; r Medical Microbiology Research Laboratory, Paulo de Góes Institute of Microbiology, Federal University of Rio de Janeiro, Rio de Janeiro, Brazil; s Laboratory of Enteropathogens, Veterinary and Food Microbiology, Biomedical Institute, Fluminense Federal University, Niterói, Brazil; t Laboratory of Applied Molecular Microbiology, Department of Clinical Analysis, Federal University of Santa Catarina, Florianópolis, Brazil; University of Arizona/Banner Health

**Keywords:** Enterobacterales, resistome, virulome, high-risk clones, One Health, multidrug resistance, carbapenems, colistin, carbapenemases, ESBL, MCR, genomic surveillance, South America

## Abstract

The dissemination of carbapenem-resistant and third generation cephalosporin-resistant pathogens is a critical issue that is no longer restricted to hospital settings. The rapid spread of critical priority pathogens in Brazil is notably worrying, considering its continental dimension, the diversity of international trade, livestock production, and human travel. We conducted a nationwide genomic investigation under a One Health perspective that included Escherichia coli strains isolated from humans and nonhuman sources, over 45 years (1974–2019). One hundred sixty-seven genomes were analyzed extracting clinically relevant information (i.e., resistome, virulome, mobilome, sequence types [STs], and phylogenomic). The endemic status of extended-spectrum β-lactamase (ESBL)-positive strains carrying a wide diversity of *bla*_CTX-M_ variants, and the growing number of colistin-resistant isolates carrying *mcr*-type genes was associated with the successful expansion of international ST10, ST38, ST115, ST131, ST354, ST410, ST648, ST517, and ST711 clones; phylogenetically related and shared between human and nonhuman hosts, and polluted aquatic environments. Otherwise, carbapenem-resistant ST48, ST90, ST155, ST167, ST224, ST349, ST457, ST648, ST707, ST744, ST774, and ST2509 clones from human host harbored *bla*_KPC-2_ and *bla*_NDM-1_ genes. A broad resistome to other clinically relevant antibiotics, hazardous heavy metals, disinfectants, and pesticides was further predicted. Wide virulome associated with invasion/adherence, exotoxin and siderophore production was related to phylogroup B2. The convergence of wide resistome and virulome has contributed to the persistence and rapid spread of international high-risk clones of critical priority E. coli at the human-animal-environmental interface, which must be considered a One Health challenge for a post-pandemic scenario.

**IMPORTANCE** A One Health approach for antimicrobial resistance must integrate whole-genome sequencing surveillance data of critical priority pathogens from human, animal and environmental sources to track hot spots and routes of transmission and developing effective prevention and control strategies. As part of the Grand Challenges Explorations: New Approaches to Characterize the Global Burden of Antimicrobial Resistance Program, we present genomic data of WHO critical priority carbapenemase-resistant, ESBL-producing, and/or colistin-resistant Escherichia coli strains isolated from humans and nonhuman sources in Brazil, a country with continental proportions and high levels of antimicrobial resistance. The present study provided evidence of epidemiological and clinical interest, highlighting that the convergence of wide virulome and resistome has contributed to the persistence and rapid spread of international high-risk clones of E. coli at the human-animal-environmental interface, which must be considered a One Health threat that requires coordinated actions to reduce its incidence in humans and nonhuman hosts.

## INTRODUCTION

Antibiotic resistance is an ever-growing threat that contributes to serious adverse consequences, such as therapeutic failure, economic burden, and increased mortality rates worldwide (1, 2). In this regard, the multisectoral (human, animal, and agriculture) overuse of important antimicrobial drugs has been considered the main driver of the multidrug resistance phenomenon (3). In an attempt to overcome this global public health challenge, the World Health Organization (WHO) has provided a red alert for “Highest Priority Critically Important Antimicrobials'' (i.e., broad-spectrum cephalosporins, carbapenems, and polymyxins), as well as insights about emergent critical-priority pathogens, including Escherichia coli (2, 4).

The epidemiological success of multidrug-resistant (MDR) E. coli has been described in a range of host/source scenarios, where high-risk pandemic lineages (e.g., ST10, ST38, ST58, ST69, ST131, ST155, ST167, ST393, ST405, ST648, and ST410) took the lead with remarkable epidemiological relevance (5–8). In this regard, genomic versatility of these clones has enhanced their pathogenicity and competence to survive for long periods (9). In addition, their ability to transfer genetic determinants by mobile genetic elements (MGEs) has been responsible for substantial contributions to spread clinically relevant resistance genes, including those encoding resistance to colistin (*mcr*), carbapenems (e.g., *bla*_KPC_, *bla*_NDM_), cephalosporins (e.g., *bla*_CTX-M_) and fluoroquinolones (e.g., *qnr*, *aac[6′]-Ib-cr*), among others (7).

To mitigate these challenges, an interdisciplinary One Health approach has been encouraged to prevent and combat the emergence and dissemination of antibiotic resistance interlinking humans, animals, and their shared environments (10). Herein, we performed a One Health surveillance using microbiological methods and whole-genome sequencing (WGS) to provide an up-to-date scenario of the epidemiology, antimicrobial-resistance profile, genomic features, phylogenetic relationship, and patterns of E. coli recovered from the human-animal-environmental interface in Brazil, the largest country in Latin America.

## RESULTS

### One Health background of E. coli strains.

E. coli genomes investigated included 167 strains from human-animal-environment interface circulating in 40 Brazilian cities, and 16 states with highest population density (North: Pará and Tocantins states; Northeast: Paraíba, Pernambuco, Ceará, Sergipe, Rio Grande do Norte and Bahia states; Midwest: Goiás and the Federal District states; Southeast: Minas Gerais, São Paulo, and Rio de Janeiro states; South: Paraná, Santa Catarina, and Rio Grande do Sul states).

These strains have been collected between 1974 and 2019, and included humans (*n =* 85), animals (*n =* 42: dog, cat, cattle, chicken/poultry, horse, turkey, anteater, elephant, fish, owl, ocelot, penguin, and vulture), food (*n =* 15: chicken, shrimp, mussels, oysters, spinach, and cabbage) and environmental (*n =* 25: freshwater, seawater, soil, drainage, and directional residue) sources (Table S1). The strains were selected according to resistance profile; included 54 genomes sequenced in this project and 113 sequences available in public databases.

**FIG 1:**
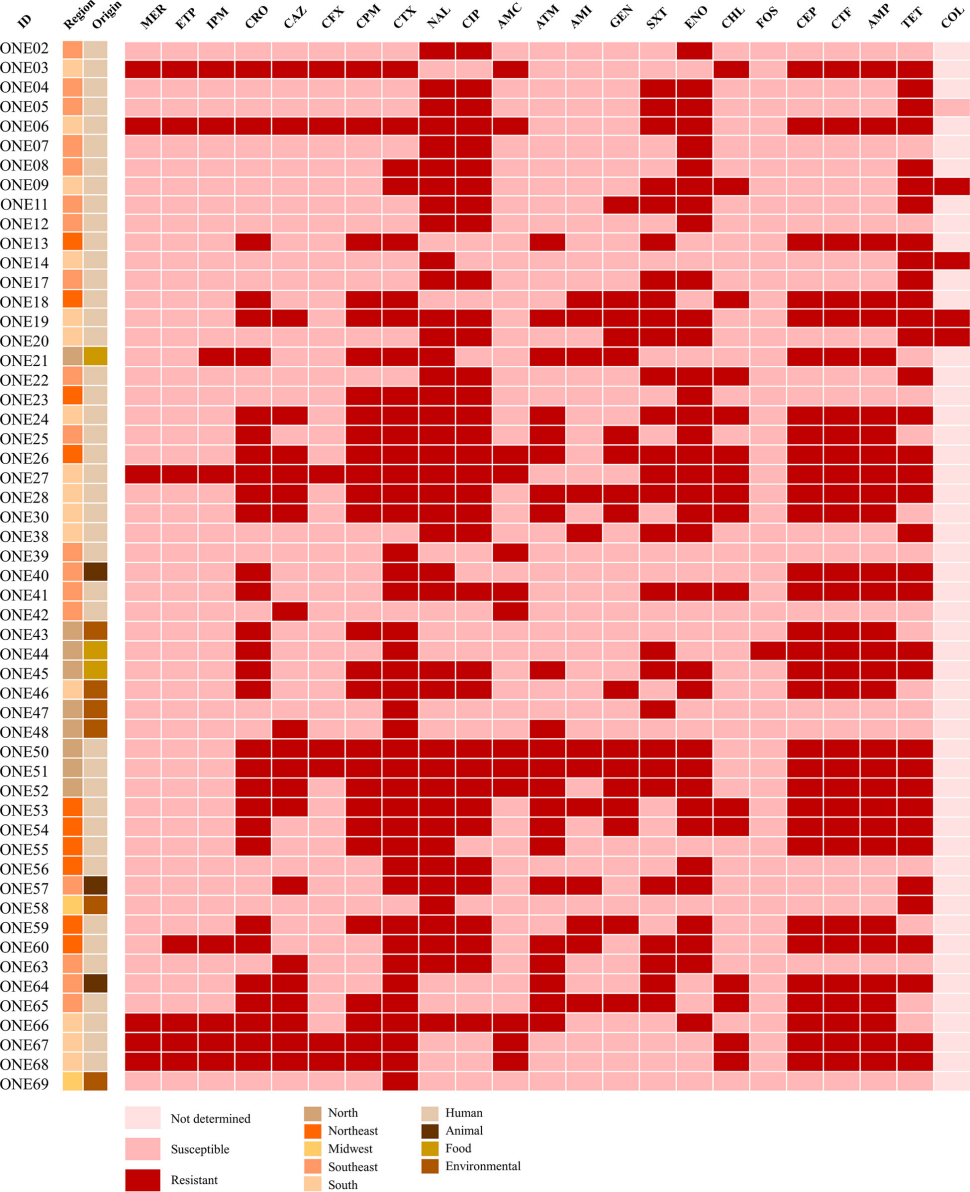
Heatmap showing the antimicrobial resistance profile of 54 Escherichia coli strains from Brazil. Boxes in dark red represent the antibiotics to which the strains exhibited a resistance profile, whereas boxes in light red represent susceptible profiles. Additionally, boxes in light pink illustrate the antibiotics that have not been tested. Antibiotic classes are abbreviated as follows: meropenem (MER), ertapenem (ETP), imipenem (IPM), ceftriaxone (CRO), ceftazidime (CAZ), cefoxitin (CFX), cefepime (CPM), cefotaxime (CTX), nalidixic acid (NAL), ciprofloxacin (CIP), amoxicillin/clavulanate (AMC), aztreonam (ATM), amikacin (AMI), gentamicin (GEN), trimethoprim-sulfamethoxazole (SXT), enrofloxacin (ENO), chloramphenicol (CHL), fosfomycin (FOS), cephalothin (CEP), ceftiofur (CTF), ampicillin (AMP), and tetracycline (TET). The colistin (COL) resistance was determined by the broth microdilution method.

The 54 E. coli strains screened in this study displayed resistance to clinically relevant fluoroquinolones, aminoglycosides, colistin, broad-spectrum cephalosporins and/or carbapenems, of which 42 were classified as multidrug-resistant strains (Fig. 1).

### E. coli clones belonging to pandemic sequence types (STs).

**FIG 2:**
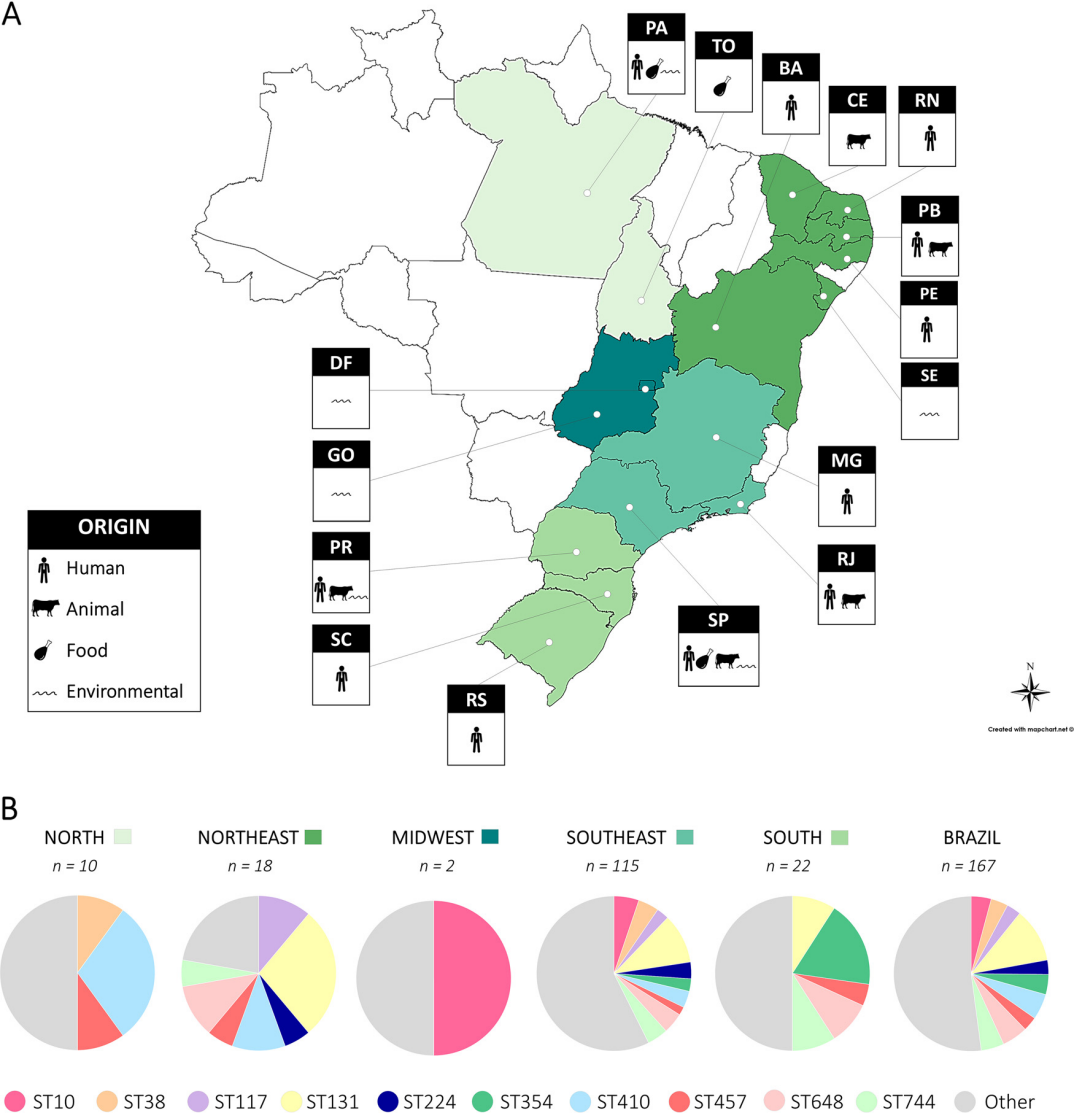
(A) Geographic distribution of Escherichia coli strains (*n =* 167) circulating at the human-animal-environmental-food interface in all regions of Brazil (North, Northeast, Midwest, South, and Southeast). North: PA (Pará), TO (Tocantins); Northeast: BA (Bahia), CE (Ceará), RN (Rio Grande do Norte), PB (Paraíba), PE (Pernambuco), SE (Sergipe); Midwest: DF (Distrito Federal), GO (Goiás); Southeast: MG (Minas Gerais), RJ (Rio de Janeiro), SP (São Paulo); and South: PR (Paraná), SC (Santa Catarina), and RS (Rio Grande do Sul). The map was created using an online service (https://mapchart.net/). (B) Representation of major international clones circulating in Brazilian regions.

Among 167 genomes of E. coli strains circulating at the human-animal-environmental-food interface (from humans; pets: dog and cat; livestock animals: cattle, chicken/poultry, horse, and turkey; wild animals: anteater, elephant, fish, ocelot, owl, penguin, and vulture; meat: chicken; crustacean: shrimp; seafood: mussels and oysters; vegetables: spinach and cabbage; freshwater; seawater; soil; drainage; and directional residue) in all regions of Brazil (Fig. 2A), we identified 69 different multilocus sequence types, in addition to 4 novels STs. The dissemination of international clones ST10, ST38, ST117, ST131, ST224, ST354, ST410, ST457, ST648, and ST744 was confirmed (Fig. 2B). Most common STs associated with human hosts were ST131, ST410, and ST354, whereas those associated with animal hosts were ST131 and ST648. On the other hand, ST38 and ST10 were the most predominant among food and environmental strains. Dominant overlaps in STs shared by all reservoirs/hosts (human, animal, food, and environmental) were associated with ST131 and ST648, which represent 11.4% (*n =* 19) and 5.4% (*n =* 9) of all strains, respectively.

### Broad resistome among MDR E. coli strains circulating at the human-animal-environment interface.

**FIG 3:**
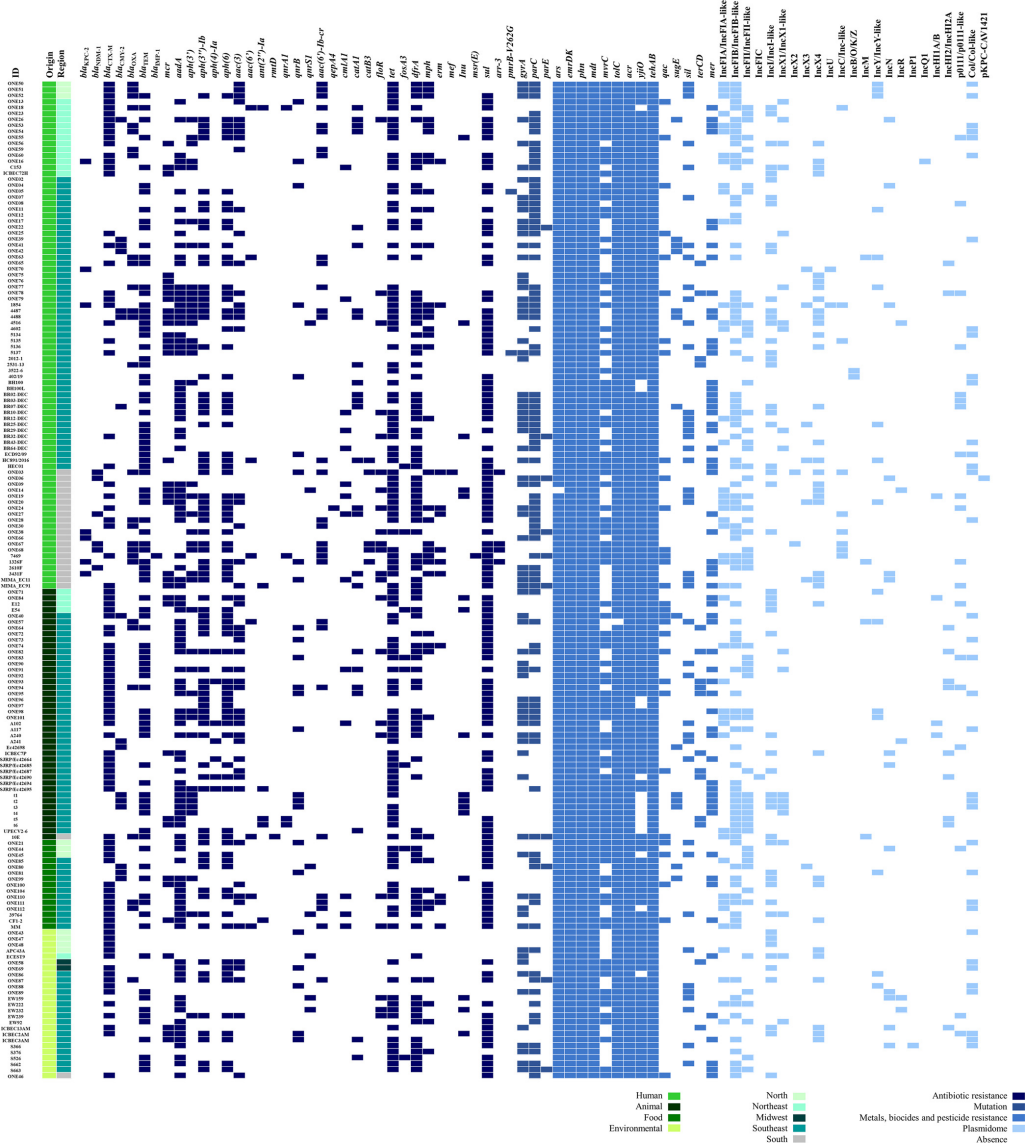
Heatmap displaying the resistome and plasmidome of Escherichia coli strains considering the ST, region, and source features. The colored regions in different shades of blue represent the presence of (1) antibiotic resistance genes (β-lactams, phenicols, colistin, tetracyclines, glycopeptides, aminoglycosides, fosfomycin, trimethoprim, macrolides, quinolones, sulfonamides, and rifampin class); (2) mutation in the quinolone resistance determining region (QRDRs) and/or *pmrB* gene; (3) metals, biocides (QACs) and pesticide (glyphosate) resistance genes; and (4) plasmid incompatibility group (Inc.); while blank fragments represent their absence.

The resistome (antibiotics, heavy metals, pesticide, and disinfectants) of the 167 E. coli analyzed is quoted in the Fig. 3. In brief, *bla*_CTX-M_- (52.7%) and *bla*_TEM_-type (53.9%) were the predominant β-lactam resistance genes, regardless of the source and origin. In this regard, a high occurrence of *bla*_CTX-M_-type genes in food (73.3%), animal (69.0%), environment (60.0%), and human samples was confirmed, with *bla*_CTX-M-2_, *bla*_CTX-M-8_ and *bla*_CTX-M-15_ variant being widely disseminated. Otherwise, *bla*_KPC-2_ (4.2%), *bla*_NDM-1_ (3.6%), and *bla*_IMP-1_ (0.6%) carbapenemase genes were only predicted in human isolates. Genes *bla*_OXA_- (17.4%) and *bla*_CMY-2_-type (10.2%) were also identified among the 167 E. coli genomes analyzed. Of concern, *mcr-*type colistin resistance genes (*mcr-1.1*, *mcr-1.5*, *mcr-5.1*, *mcr-5.3*, and *mcr-9* variants) were detected in 35 E. coli genomes (21.0%) of human, animal, environmental and food isolates.

The presence of plasmid-mediated quinolone resistance (PMQR) [*qnrA* (2.4%), *qnrB1* (1.2%), *qnrB2* (2.4%), *qnrB19* (11.4%), *qnrS* (4.2%)], *aac(6′)-Ib-cr* (17.9%), and *qepA4* (0.6%) genes was also predicted. Although the *aac(6′)-Ib-cr* gene was mostly found in isolates from human hosts (70%), it was also detected in animal (13.3%), food (13.3%), and environmental (3.3%) E. coli genomes. The *qnrB* genes were present in E. coli genomes from all ecological scenarios surveyed, whereas *qepA4* was only found in human isolates. Quinolone resistance was also mediated by mutations (*gyrA*-S83L, *gyrA*-D87N, *gyrA*-D87Y, *gyrA*-D87G, *gyrA*-D87V, *parC*-S80I, *parC*-A56T, *parC*-S80R, *parC*-E84K, *parC*-E84V, *parC*-E84G, *parE*-S458A, *parE*-S458T, *parE*-I355T) in the quinolone resistance determining region (QRDRs), in both human and nonhuman strains.

Aminoglycoside [*aad*, *aph(3′)*, *aph(3”)-Ib*, *aph*(4*)-Ia*, *aph*(6), *aac*(3), *aac(6’)*, *ant(2”)-Ia*, and *rmtD*], trimethoprim (*drfA*), tetracycline (*tet*), fosfomycin (*fosA3*), phenicol (*cmlA1*, *catA1*, *catB3*, and *floR*), sulfonamide (*sul*), macrolide *(mph, erm, mef, inu, and msr[E])*, and rifampicin (*arr-3*) resistance genes were also predicted, as summarized in Fig. 3.

Genes conferring resistance to heavy metals (arsenic, *n =* 166/167; silver, *n =* 42/167; mercury, *n =* 60/167; and tellurium, *n =* 16/167), QAC disinfectants (*n =* 167/167), and pesticide (glyphosate, *n =* 167/167) were identified in E. coli isolates from all surveyed hosts and reservoirs. In contrast, tellurium resistance genes (*terC* and/or *terD*) were not identified in food strains, whereas no lead resistance genes were detected in this study. Detailed resistome information is also shown in Table S1.

### Mobilome analysis.

**FIG 4:**
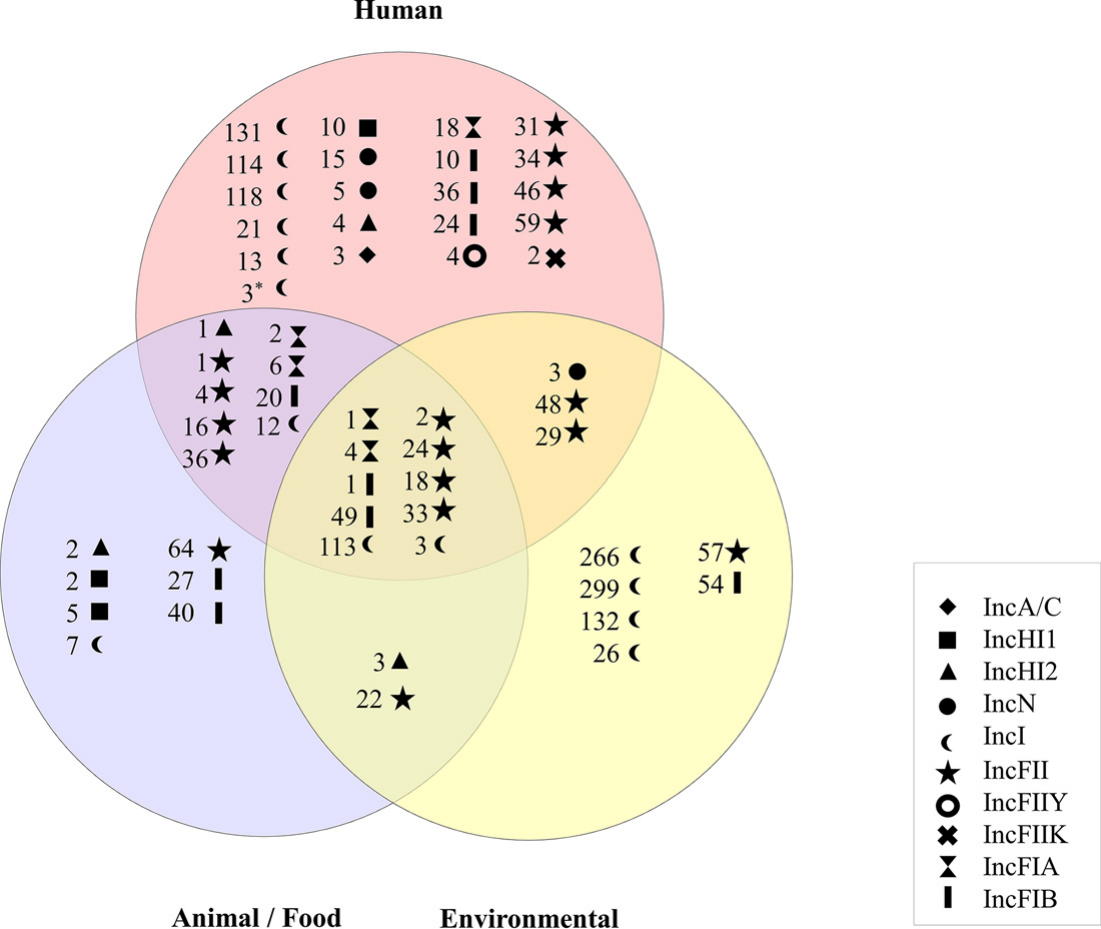
Venn diagram displaying plasmidial MLST (pMLST) dynamics in the One Health context.

The investigation using CGE - Center for Genome Epidemiology - tool, showed that the plasmid population of E. coli lineages was diverse, with the most common incompatibility group being IncFIB/IncFIB-like (52%), followed by IncFII/IncFII-like (33.5%), IncI/IncI-like (31.1%), IncFIA/IncFIA-like (*n =* 25.7%), Col/Col-like (21.5%), and IncX4 (20.9%). Additionally, 129 (77.2%) E. coli genomes presented more than one plasmid replicon type. E. coli genomic sequences that presented IncF (FAB type), IncI1, IncN, IncA/C, IncHI1, and IncHI2 replicons were also evaluated for plasmid MLST (pMLST), as shown in Fig. 4. We observed specific pMLST allele sequences according to the bacterial source. On the other hand, some subtypes (A1 and A4 allelic variant from IncFIA plasmid; B1 and B49 from IncFIB plasmid; F2, F24, F18, F33 from IncFII plasmid; 113 and 3 from IncI plasmid) were found regardless of the source of origin.

### Virulome analysis.

**FIG 5:**
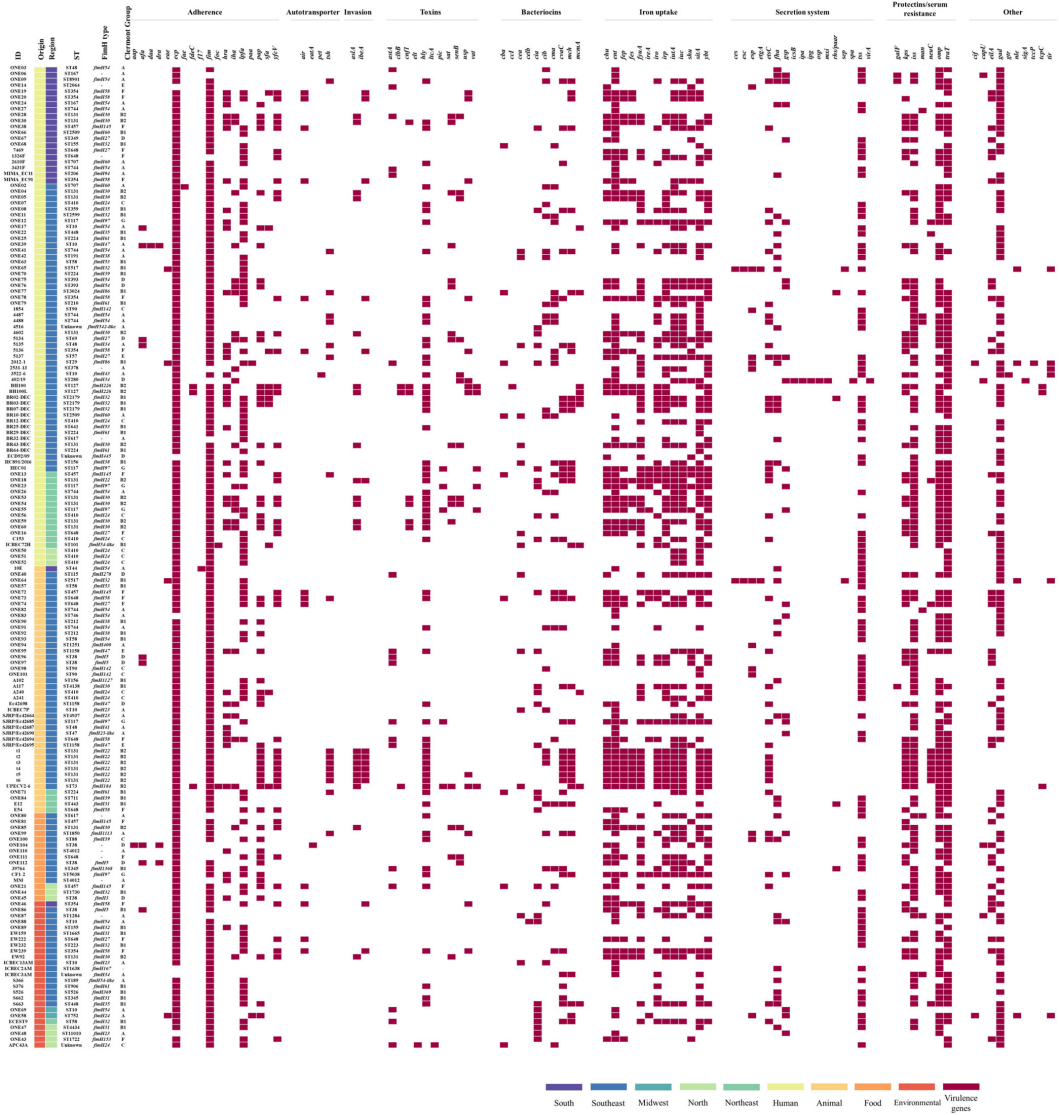
Schematic representation of virulome, sequence type, *fimH* type, and Clermont phylogroup of Brazilian Escherichia coli strains indicated as per the legend. Not determined (-).

The virulome context of E. coli strains is quoted in Fig. 5. In an effort to better understand the virulome scenario, the genes were grouped into the following key components: (i) adherence, (ii) autotransporter, (iii) invasion, (iv) toxins, (v) bacteriocins, (vi) iron uptake, (vii) secretion systems, (viii) protectins/serum resistance, and (ix) other factors of pathogenicity. The predominant adherence-associated genes were *ecp* (96.5%), *fim* (93.4%), and *lpfA* (52.7%), whereas genes encoding toxins were *hly* (37.7%), *astA* (10.8%) and *sat* (9.6%). The main genes involved in protectins/serum resistance were *traT* (73.1%), *omp* (71.3%) and *iss* (64.7%); and the identified iron acquisition system (siderophores) genes were *ent* (65.3%), *sitA* (55.7%), *iutA* (47.3%), *iucC* (46.1%), *ybt* (43.1%), *fyuA* (43.1%), and *irp* (41.9%). For the secretion system, the presence of the *tss* gene was highlighted, whereas the *gad* (glutamate decarboxylase) gene, which contribute to acid resistance was also identified. Others virulence genes, includying *aap*, *daa*, *fae*, *f17*, *eatA*, *pet*, *ltcA*, *elt*, *ccI*, *celb*, *icsB*, *ipa*, *ipg*, *osp*, *mxi*, *spa*, *virA*, *gtr*, *sigA*, and *tccp* occurred only in single strains (Table S1).

An extensive virulome (≥90 virulence genes) was predicted in genomes of lineages ST127 (phylogroup B2, O6:H131) and ST73 (phylogroup B2, O6:H1), identified in human and animal hosts. Strains collected before the 2000s (2012-1, 2531-13, 3522-6, BH100, and BH100L), showed broad virulome (on average 58 genes), and resistance genes to β-lactam (*bla*_TEM_, *bla*_OXA_), phenicol (*catA1*), tetracycline (*tet[B]*), trimethoprim (*dfrA*), sulfonamide (*sul1*), and aminoglycoside (*aadA1* and *aph[3′]-Ia*).

Regarding classification of type 1 fimbria (*fimH*) and serotyping, 38 distinct *fim* types and diverse serotypes were found, where Clermont typing showed that phylogroups A (25.7%) and B1 (25.1%) were predominant (Fig. 5).

### Phylogenomics and evolutionary dynamics of human and nonhuman E. coli clones.

**FIG 6:**
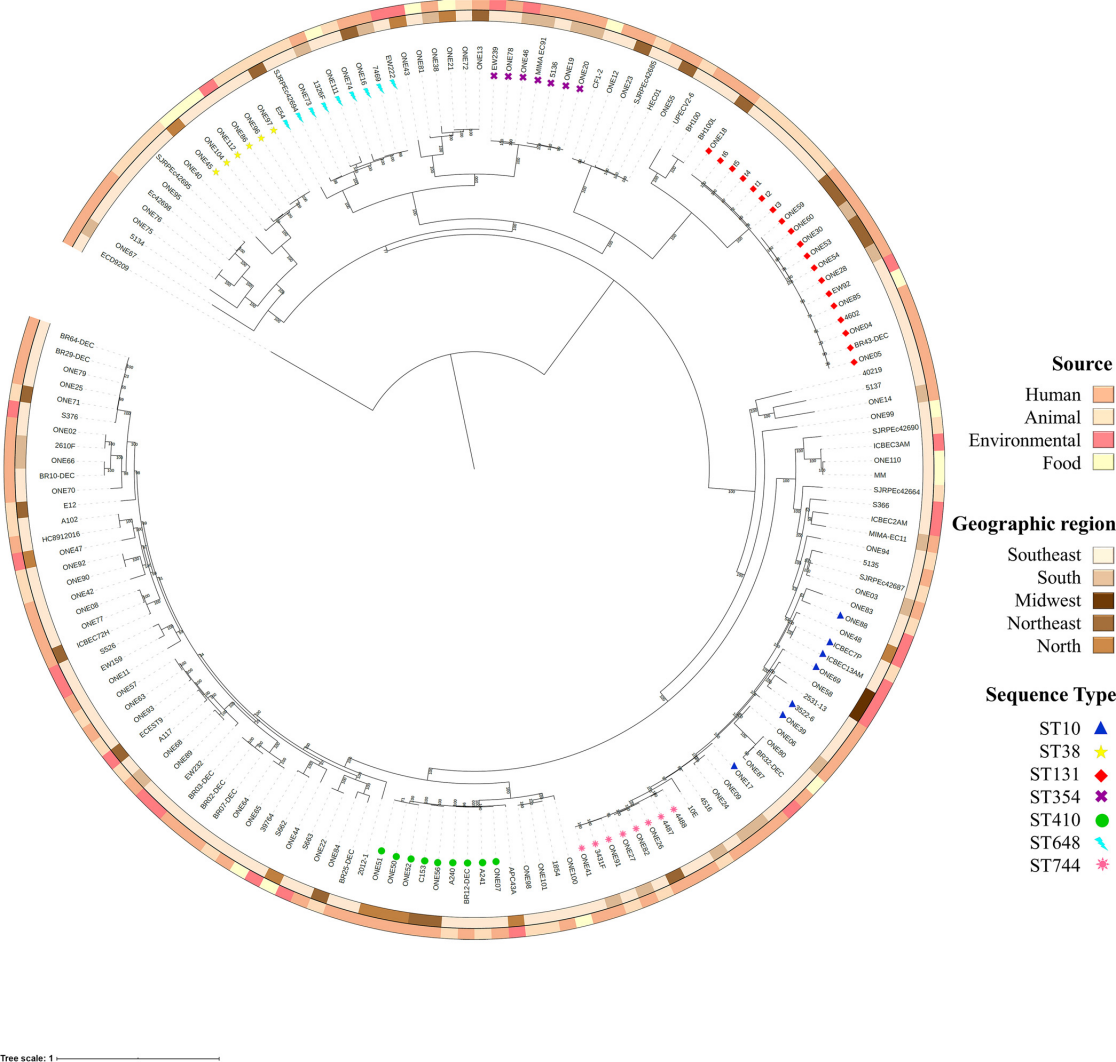
Phylogenetic tree based on the core genome (1793 genes) of the 167 Brazilian Escherichia coli strains circulating at the human-animal-environmental-food interface. The figure was generated with iTOL version 5.6.1 (https://itol.embl.de), and the interactive version of this tree can be found at https://microreact.org/project/noM6Wi46mnpdYSzENnWmKX. Phylogenetic tree was rooted at midpoint. The ST10 clade includes isolates belonging to ST10 (*adk*-10, *fumC*-11, *gyrB*-4, *icd*-8, *mdh*-8, *purA*-8, *recA*-2), the closely single locus variants (SLV) ST11010 (*icd*-1290), ST752 (*recA*-49) and ST167 (*purA*-13), and double locus variants (DLV) ST378 (*icd*-1, *purA*-66) and ST617 (*purA*-8, *recA*-73).

The SNP maximum likelihood tree of human and nonhuman E. coli genomes, constructed using RAxML-NG (100 bts), is presented in Fig. 6. Strikingly, E. coli lineages from distinct sources (human, animal, environmental and food) and geographic regions, in Brazil, were closely related in the phylogenetic tree, being grouped in clades. Pangenome and SNP matrix data are quoted in Fig. S1 and, Table S2 and S3.

**FIG 7:**
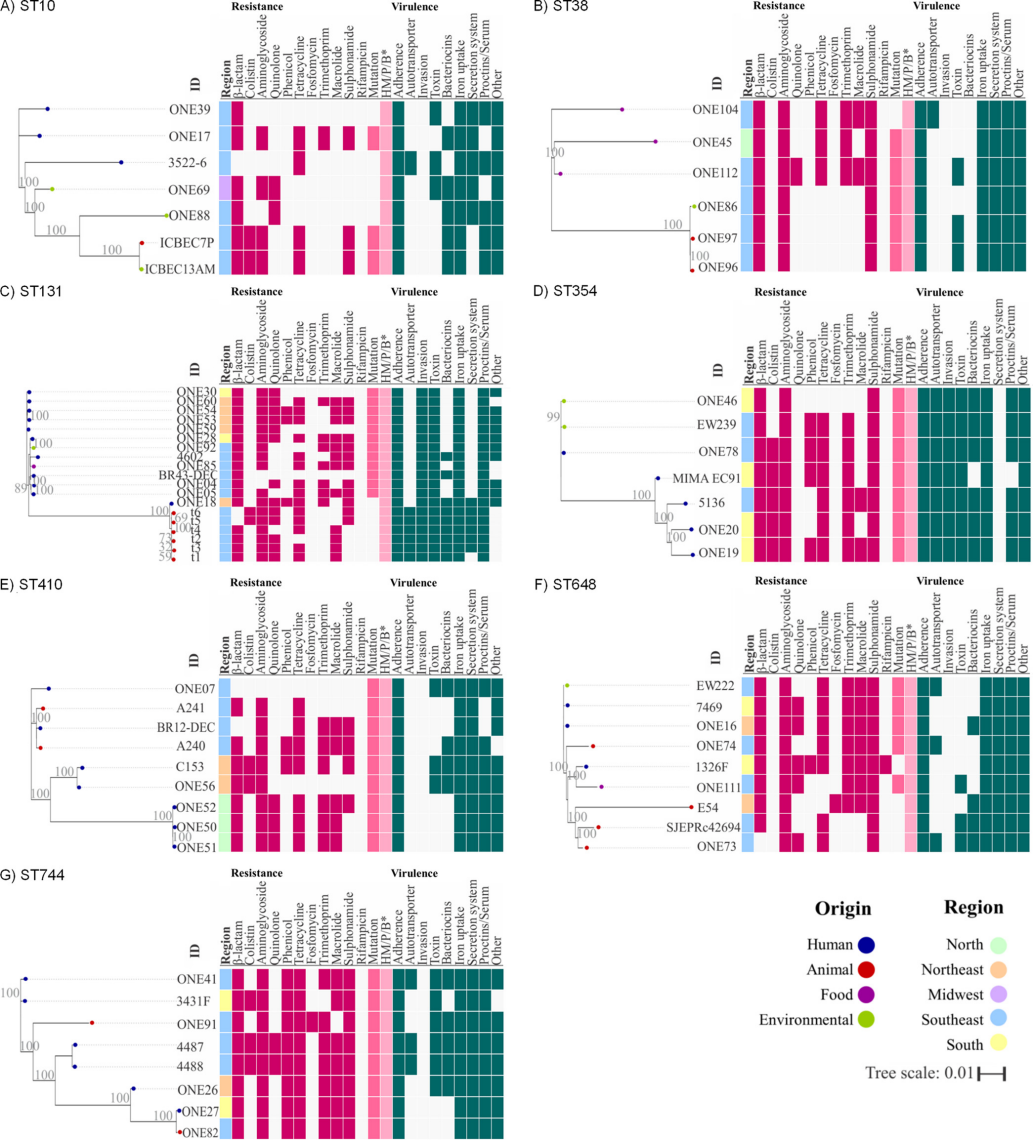
Zoom-in depicting a maximum-likelihood (ML) phylogenetic tree built using core genome single-nucleotide polymorphisms (SNPs) of E. coli lineages (A-ST10, B-ST38, C-ST131, D-ST354, E-ST410, F-ST648, and G-ST744). The phylogeny was tested against 100 bootstrap replications and the image was visualized using iTOL version 5.6.1 (https://itol.embl.de). The panel on the right represents the geographic region and the resistome and virulome context.

A zoom-in on the representative clades of important pandemic clones depicted in the phylogenetic tree is shown in Fig. 7. In this regard, E. coli strains belonging to the ST10, collected in the Southeast and Midwest regions of Brazil, were nested within a human, animal, and environmental clade. ST10 has been a persistent One Health clone, present in this country since at least 1989. The ST10 clade included strains carrying *bla*_CTX-M-8_ (ONE69 and ONE88), *bla*_CTX-M-1_ and *mcr-1.1* (ICBEC7P and ICBEC13AM), and *bla*_CMY-2_ (ONE39) resistance genes (Fig. 7A and Table S3).

For the ST38 clone, phylogenetic tree revealed a clade that included strains isolated from food (mussels, chicken meat, cabbage), birds of prey (vulture), and environmental (seawater) samples (Fig. 7B). ST38 has been present since at least 2016, at the Southeast and North regions of the country. Specifically E. coli strains ONE97 and ONE96, isolated from birds of prey in 2017, were closely related (>98% identity) to the environmental ONE86 strain isolated in 2016, from a seawater sample at the same region; and carried an identical resistome, including the *bla*_CTX-M-14_ extended-spectrum β-lactamase (ESBL)-gene.

For the international ST131 clade, most strains presented a broad and critical resistome that included *bla*_CTX-M-8_, *bla*_CTX-M-9_, *bla*_CTX-M-15_ and *bla*_CTX-M-27_ ESBL genes. These strains have been identified from 2010, highlighting the rapid dissemination of this international high-risk clone over the last decade (Fig. 7C, Table S3). A multiplicity of origins and geographic features among these strains denoted One Health implications. All strains displayed a virulome contributing to adherence, invasion, toxins production, iron uptake, and protectins/serum resistance.

The phylogenomic analyses of ST354 showed a clade with strains presenting the broadest set of virulence determinants, which have been circulating at the human-environmental interface from 2016 (Fig. 7D and Table S3). From the resistome perspective, all human E. coli ST354 strains carried the *mcr-1.1* gene; with ONE46, EW239 and ONE78 strains being more closely related (>99% identity).

Lineages of ST410 have been identified at the human-animal interface in North, Northeast and Southeast regions of Brazil, from 2014. Phylogenomics revealed that while ONE50, ONE51 and ONE52 human strains shared 99.9% identity (Fig. 7E and Table S3) and an identical resistome and virulome, A240 and A241 (animal hosts) and BR12-DEC (human host) strains shared >93% identity.

Phylogenomic investigation also confirmed that the high-risk ST648 clone has been another closely related pandemic lineage with One Health implications in this country (Fig. 7F), from at least 2013, when the first *bla*_KPC-2_-positive E. coli strain (1326F) belonging to this clone was isolated from a human host in Southern Brazil (Table S3). In the phylogenetic three, the 1326F strain was closely related to the ONE111 *bla*_CTX-M-15_-positive E. coli strain isolated from a food sample collected in 2016, in the Southeast region. Moreover, from core genome SNPs analysis, WHO critical priority EW222 (*bla*_CTX-M-15_), 7469 (*bla*_CTX-M-15_, and *bla*_IMP-1_) and ONE16 (*bla*_CTX-M-15_, and *bla*_KPC-2_) strains isolated from environmental and human samples collected in the Northeast, Southeast and South regions, shared >99% identity (Fig. 7F and Table S3).

Finally, E. coli strains belonging to ST744 were clustered within a human-animal clade that included stains circulating in different geographical regions, since at least 2014 (Fig. 7G and Table S3). In this regard, the first strain belonging to this lineage was isolated from a human host and carried *bla*_KPC-2_ and *mcr1.1* genes, being closely related to the ONE41 (*bla*_CTX-M-1_- and *bla*_CMY-2_-positive) human strain identified in 2015, at the Southeast region. Interestingly, ONE27 (*bla*_NDM-1_-positive) and ONE82 (*bla*_CTX-M-2_-positive) strains were isolated from human and a fish samples at the South and Southeast regions, respectively; sharing 98.1% identity in the phylogenetic three (Fig. 7G).

## DISCUSSION

Brazil is the largest and most populated country in South America, with a wide geographical area that comprises different ecosystems with a remarkable biodiversity (11). Unfortunately, in most metropolitan areas of this country antimicrobial resistance has been a critical issue, not restricted to hospital settings (5). In fact, in the last years, there has been growing concern that the nationwide propagation of antibiotic resistance is also associated with environmental reservoirs that are linked to anthropogenic activities such as animal husbandry, agribusiness activities and wastewater treatment. In this study, we performed a genomic-based surveillance using a One Health approach, elucidating the genomic background of E. coli strains recovered from the human-animal-food-environmental interface over the past 45 years. Our findings highlight (i) a broad antimicrobial resistome, (ii) convergence of virulence and resistance genes, (iii) successful expansion of international high-risk clones, and (iv) phylogenomic diversity with strains isolated from human and nonhuman samples clustering together.

The resistome suggests that ESBL endemicity has been associated with β-lactamases conferring resistance to cefoxitin, where CTX-M-positive E. coli have been identified in humans, vegetables, chicken meat, wild and food-producing animals, pets, Amazonian fish, and aquatic environments (12–19). In our study, we found the predominance of *bla*_CTX-M-15_, followed by *bla*_CTX-M-2_, and *bla*_CTX-M-8_ ESBL genes. Interestingly, food samples were identified as potential reservoirs associated with *bla*_CTX-M_-type genes, whereas it is well documented that food-producing animals play an important role as a reservoir of MDR pathogens (20). In line with this, the hypothesis has been suggested that the commercial chicken meat could be a reservoir of E. coli strains co-harboring *bla*_CTX-M_ and colistin resistance *mcr-1* genes (21).

The overuse of colistin as a growth promoter in food-producing animals and its clinical usage to treat carbapenem-resistant infections has been a red alert to global health (22–24). We found several strains from all sources (human, animal, food, and environmental) surveyed harboring *mcr*-type genes, including those belonging to international sequence types ST10, ST131, ST354, ST393, ST410, and ST744. Main plasmids that have driven the spread of the *mcr-1* genes have belonged to IncX4, IncI2, and IncHI2 incompatibility groups (23, 25). Here, the presence of IncX4 plasmid replicon gene was confirmed in 82.8% of *mcr-*positive strains, which reinforces the endemicity of this plasmid type within a One Health perspective.

The rapid spread of *bla*_KPC-2_-positive carbapenem-resistant E. coli strains during the last years is another epidemiological data observed in this study, which also requires closer attention, particularly due to its association with international high-risk clones ST90, ST224, ST648 and ST744 (5, 7, 26). Although, there has been concern around *bla*_KPC_ in E. coli of ST131, another globally disseminated and clinically successful clone (27), here this lineage was only associated with the presence of *bla*_CTX-M-8_, *bla*_CTX-M-9_, *bla*_CTX-M-15_, *bla*_CTX-M-27_ and *bla*_CMY-2_ β-lactamase genes.

Another point that deserves attention is the rate of quinolone resistance related to chromosomal mutations and/or PMQR genes. Quinolone resistance has not been limited to health care-associated infections. In this study, approximately 37% of strains, including those from human, animal, food, and environmental sources/host presented one or more quinolone resistance genes, whereas 64% of the total had at least one mutation, confirming a worldwide trend, where the presence of quinolone-resistant E. coli strains has been found in a wide range of human and nonhuman reservoirs (5, 7, 27–32).

Our findings also reveal that the resistome of the screened E. coli strains extends to heavy metals, biocides, and pesticides, which could contribute for the development of coresistance to antibiotics and other antimicrobial agents (33). This should be considered a critical problem since biocides are agents for disinfection commonly used in domiciliary, veterinary and hospital settings (33). Common active ingredients in disinfectants are quaternary ammonium compounds (QACs), of which benzalkonium chloride is a concern because of its widespread use combined with environmental impacts (34). Disinfectants containing quaternary ammonium compounds, including benzalkonium chloride (BAC), act mainly by disturbing the integrity and function of the cell membrane that leads to cell death. Since bacterial resistance to BAC compounds is based on overexpression of efflux pumps, QAC-induced overexpression of efflux pumps can lead to: i) cross-resistance for clinically relevant antibacterial agents, including fluoroquinolones; ii) stress response facilitating mutation in the Quinolone Resistance Determining Region; iii) biofilm formation increasing the risk of transfer of mobile genetic elements carrying fluoroquinolone or QAC resistance determinants (35). Once benzalkonium chloride enters the environment, it can be lethal to aquatic organisms contributing to antimicrobial resistance. As well as for antibiotic residues, environmental entry of QACs is through wastewater effluent and sewage. Therefore, the dissemination of antibiotic resistance and *qac* genes among E. coli strains circulating across clinical boundaries could be a direct consequence of anthropogenic activities (36).

Convergence of resistence and virulence in E. coli lineages is another issue that has worried the medical community (37). Indeed, virulence potential of ESBL- and/or AmpC-β-lactamase-producing E. coli strains from healthy food animals from Europe and South America has been recently documented (38–39). In our study, virulence factors involved in adherence and biofilm (e.g., *afaA*, *faeC*, *fimC*, *focX*, *hra*, *iha*, *pap*, and/or *sfa* genes) were remarkably shared, mainly by the international high-risk clones identified. Previous analysis on the distribution of adherence/biofilm genes in E. coli lineages has highlighted the presence of these genes in strains belonging to ST131 and ST648 clones (40).

Regarding protectins/serum resistance genes, most E. coli strains (59.9%) were positive for *traT*, *omp*, and *iss* genes. The *iss* gene plays a role on microbial survival to serum due to the phagocytosis protection factor; *omp* (outer membrane protein) gene is associated with evasion of the body's defense allowing intracellular survival, whereas *traT* gene is involved in the inhibition of complement system activity (41).

Siderophores (high-affinity iron chelating molecules) have been received special attention as essential virulence factors of bacteria, acting as a toxin and/or modulator of the immune system of the host (42–45). Strains that can produce more siderophores are considered highly virulent (45). In this study, more than 40% E. coli strains also carried siderophore (aerobactin, yersiniabactin, salmochelin, and enterobactin)-encoding genes. Additionally, we predicted clinically relevant virulence genes encoding hemolysin, enteroaggregative heat-stable toxin, secreted autotransporter toxin, cytotoxic necrotizing factor 1, serine protease autotransporter, and vacuolating autotransporter toxin (41, 46).

E. coli have been grouped into eight major phylogroups (i.e., A, B1, B2, C, D, E, F and G) based on genetic analyses. While A and B1 phylogroups are widely associated with commensal lineages, B2 has been a predominat pathogenic lineage. Additionally, strains from phylogroup B1 have been found to persist longer and to tolerate lower temperatures than the remaining phylogroups, resulting in higher ability to colonize aquatic ecosystems than A and B2 phylogroups, which have been linked to an animal-associated lifestyle (47). Many recognized virulence factors are enriched among lineages belonging to specific phylogroups of E. coli that are evolutionarily quite old, globally distributed, and commonly inhabiting healthy human guts, such as phylogroup B2. In this study, Clermont phylotyping analysis confirmed that strains carrying the colibactin gene *clbB* belong to phylogroup B2, as previously reported (48). On the other hand, some lineages of E. coli B2 carried the broadest virulome. In this way, it has been suggested that strains B2 have been commonly responsible for extraintestinal infections and possess numerous virulence genes (17, 37, 41). The occurrence of clinically relevant resistance genes (e.g., *bla*_CTX-M_ and *bla*_KPC_) was observed in strains belonging to A and B1 phylogroups, which presented a smaller set of virulence genes, as previously reported (37, 41, 49, 50).

Among international clones predicted ST131-B2 presented *fimH30* and *fimH22* alleles. The *fimH30* appears to be the most prevalent in E. coli ST131 (51, 52), whereas *fimH22* type has been observed in strains from animal populations, increasing the risk of zoonotic transmissions (52). Additionally, 68 different STs were identified. The ST131, ST410 and ST354 were the most common STs associated with a human origin, whereas for animal hosts, ST131 and ST648 were predominant. Our findings confirm previous studies showing predominance of ST10, ST131, ST410, ST648, and ST744 in MDR E. coli strains from human and/or animal clinical samples (7). In Brazil, E. coli belonging to ST131, ST10, ST69, ST73, ST354, ST405, and ST648 have been reported from human samples (53–54), and ST73, ST10, ST131, ST648 have been recovered from captive and wild birds, poultry, and cats (5, 55, 56). Finally, we identified the ST10 as the predominant ST found in environmental samples, as previously reported in European and Asian countries (57–60). Previous genomic studies have demonstrated a phylogenomic relatedness of ST131, ST58, ST10, ST648 and ST38 from Brazil with international clones of the same STs, denoting successful adaptation of international clones of E. coli at the human-animal-environment interface, in Brazil (5, 61–65).

The establishment of high-risk clones overlapping human and nonhuman sources offers the opportunity to successfully disseminate resistance genes that makes difficult the control this situation (9, 10, 60). Under this perspective, other factors that drive antibiotic resistance deserve attention, such as overuse of antibiotics in multiple sectors to treat infections or for agriculture purposes, poor implementation of hygiene and sanitization actions, and the environmental contamination by inappropriate waste treatment (3). These aspects provide a snapshot of the need to enforce a comprehensive One Health strategy that considers the connection of human, animal, and environmental health in order to preserve the effectiveness of currently available antibiotics (3).

In general, over the last decades, there has been a significant increase in antibiotic prescribing and consumption leading bacterial resistance to the point of becoming a global priority (66). During COVID-19 pandemic use of antibiotics and biocide has grown even more rapidly (66, 67), which can strongly favor the selection and dissemination of WHO critical priority resistant pathogens at the human-animal interface (68). In fact, an increase in the incidence of antimicrobial resistance has been documented during COVID-19 pandemic (69, 70), with a rapid increase in multidrug-resistant organisms, including extended-spectrum β-lactamase (ESBL)-producing and/or carbapenem-resistant NDM-producing Enterobacterales, A. baumannii, and methicillin-resistant *Staphylococcus aureus* (MRSA). The cause has been multifactorial and is particularly related to high rates of antimicrobial agent utilization in COVID-19 patients with a relatively low rate of co- or secondary infection (71). In this regard, household transmission of carbapenemase-producing organisms has been linked to hospital discharge (72), becoming a potential way for transmission of such bacteria to humans and companion animals during the pandemic period (73); since COVID-19 pandemic has increased relationships and interactions between family members, and between humans and pets, supported by widespread social distancing and isolation measures. In Brazil, these facts are particularly worrying, since the prevalence of critical priority ESBL and carbapenemase producers has been higher than reported in other countries (74, 75). Additionally, in October 2021 an epidemiological alert on emergence and increase of new combinations of carbapenemases in Enterobacterales, triggered by the increased use of broad-spectrum antibiotics in patients with COVID-19, in Latin America and the Caribbean was announced by the Pan American Health Organization (PHAO), emphasizing the importance of appropriate microbiological diagnosis and the effective and articulated implementation of infection prevention and control programs (76).

As a limitation of this study, since short-read sequencing technology was used, it could influence the number of contigs and lead to the missing of resistance, virulence, and plasmid replicon genes in the genomes analyses. In addition, draft genomes may overestimate the number of accessory genes in Roary. We also did not perform a temporal analysis since it was not possible to retrieve strains from every year. Furthermore, we recognized that our study showed an uneven geographic distribution of the analyzed strains because most of them are from the Southeastern region.

In conclusion, we have investigated the genomic background of critical priority E. coli strains circulating at the human-animal-environment interface in Brazil, documenting the successful spread of international high-risk clones with a broad antimicrobial resistome, with *bla*_CTX-M_ ESBL and *mcr-1* genes being endemic, and the rapid and worrisome expansion of *bla*_KPC-2_ and *bla*_NDM-1_ carbapenemase genes. We also found clinically relevant virulomes among E. coli strains, which together with the broad resistomes could contribute to the pathogenicity. This genetic background of E. coli must be a key factor that has contributed to adaptation and dissemination of critical priority clones in human and nonhuman hosts, which is a serious problem that needs urgent actions that includes both stricter surveillance and more judicious use of antimicrobials, under a One Health perspective. Finally, these observations alert us to the worsening of the antimicrobial resistance problem in Brazil, after the COVID-19 pandemic.

## MATERIALS AND METHODS

### Sequenced isolates and metadata.

During a multicentric surveillance study (One Health Brazilian Resistance [OneBR] project, http://www.onehealthbr.com/) conducted to characterize the burden of antimicrobial resistance associated with WHO critical priority pathogens in Brazil, we collected 104 E. coli strains isolated from different sources (humans, food-producing animals, companion animals, wildlife, polluted environments, and food), over a 10-year period (2010 to 2019). The isolates were obtained from all geographic regions (North, Northeast, Midwest, Southeast and South). From this collection, 54 E. coli strains displaying resistance to broad-spectrum cephalosporins, carbapenems, colistin and/or fluoroquinolones, were selected for WGS and investigated in this study.

For comparative genomic analysis, we used 36 E. coli genomes previously published and deposited in our platform OneBR (http://www.onehealthbr.com/). Additionally, we included 77 publicly available Brazilian E. coli genomes deposited at the GenBank database (https://www.ncbi.nlm.nih.gov/genbank/). These genomes were chosen based on the resistance profile of each strain, and complete epidemiological information (i.e., source, origin, city, and date of collection). In total, 167 genomes of E. coli strains circulating at the human-animal-environment interface, in Brazil, were analyzed in this study. Detailed information is presented in Table S1.

The genomes were clustered in four groups according to their origin (human, animal, environmental, and food). The human group included samples from blood, bone fragment, catheter, feces, urine, fistula fluid, ileum mucosa, prosthesis, rectal swab, and other body fluids (e.g., foot, pelvic, or tracheal secretion). Genomes of E. coli isolated from pets (dog and cat), livestock animals (cattle, chicken/poultry, horse, and turkey), and wild animals (anteater, elephant, fish, ocelot, owl, penguin, and vulture) have been obtained from rectal/cloacal sample or different sites of infection. The food group included strains from meat (chicken), crustacean (shrimp), seafood (mussels and oysters), and vegetables (spinach and cabbage); whereas E. coli strains recovered from freshwater, seawater, soil, drainage, and directional residue, were included in the environmental group.

### Antimicrobial resistance profile.

The Escherichia coli isolates (*n =* 104) collected in this study were identified by matrix-assisted laser desorption ionization-time of flight mass spectrometry (MALDI-TOF), and screened to select MDR profiles (77, 78). For the latter, initially an overnight culture of each strain was grown on MacConkey agar plates, in the absence and presence of ceftriaxone (2 μg/mL) or colistin (2 μg/mL), at 37°C. Growing colonies were then subjected to antimicrobial susceptibility tests by disk diffusion method using human and veterinary antibiotics, which included ampicillin, amoxicillin/clavulanate, aztreonam, cephalothin, ceftriaxone, cefotaxime, ceftiofur, ceftazidime, cefoxitin, cefepime, ertapenem, imipenem, meropenem, nalidixic acid, ciprofloxacin, enrofloxacin, amikacin, gentamicin, trimethoprim-sulfamethoxazole, chloramphenicol, fosfomycin, and tetracycline. For colistin, susceptibility was determined by the broth microdilution method. Interpretative criteria were based on CLSI and/or EUCAST guidelines (79–81).

### Whole-genome sequencing.

The genomic DNA of E. coli (*n =* 54) strains was sequenced using the Illumina NextSeq (San Diego, USA) platform. In brief, single colonies of each strain were grown in 3 mL of lysogeny broth for 18 h at 37°C, and the DNA was extracted using a PureLink quick gel extraction kit (Life Technologies, CA). NextSeq libraries were constructed using the Nextera DNA Flex library prep (Illumina Inc., San Diego, CA) and sequenced using 2 × 75 bp paired-end reads. Raw sequencing data were quality filtered to remove low-quality bases (Phred quality <20) using TrimGalore v0.6.5 (https://github.com/FelixKrueger/TrimGalore) or Trimmomatic v0.32 (https://github.com/timflutre/trimmomatic). Quality-filtered reads were *De novo* assembled using Unicycler v0.4.8 (https://github.com/rrwick/Unicycler) assembler with default parameters (82). Contigs with less than 200-bp long were removed from the genome.

### Genome data analysis.

Genomic analyses of sequenced E. coli strains, and publicly available genomes (*n =* 113) were performed using the ResFinder v.4.1, VirulenceFinder v2.0, KmerFinder v3.1, PlasmidFinder v.2.0, pMLST v2.0, MLST v2.0, SerotypeFinder v2.0 and FimTyper v1.0 tools from CGE (http://genomicepidemiology.org/) (83–88). ABRicate v0.9.8 (https://github.com/tseemann/abricate) was also used to predict virulence genes profiling through the VFDB database (https://github.com/haruosuz/vfdb). Threshold ID and minimum length values (identity and coverage) of ≥90% were used for all database scanning. Plasmids with identity greater than 98% were categorized into different incompatibility (Inc.) groups. The E. coli phylogroup was performed using ClermonTyping v1.4.0 (89). Heavy metal (HM, arsenic, silver, tellurium, lead, and mercury) and disinfectants (QACs) resistance genes were screened using the BacMet2 database (http://bacmet.biomedicine.gu.se) and ABRicate v0.9.8. Additionally, pesticide (glyphosate) resistance genes were identified by *in silico* comparative analysis using an in-house database.

The genomes submitted to NCBI were annotated using the NCBI Prokaryotic Genome Annotation Pipeline v.3.2 (http://www.ncbi.nlm.nih.gov/genome/annotation_prok/).

To assess the phylogenomic relationship of E. coli strains under a One Health approach, 167 draft genomes from human and nonhumans strains, collected in several regions of Brazil, were analyzed. The analysis of the E. coli pangenome was accessed by Roary pipeline version 3.13.0 (90) using annotated draft assemblies in GFF3 format produced by Prokka (91). The gene presence/absence output from Roary was used to construct the pangenome gene presence-absence matrix that was visualized using Roary.plots.py program. The core gene alignment was used to construct the phylogenetic structure of E. coli strains. SNPs (single nucleotide polymorphism) were extracted from the core gene alignment using SNP-sites (92) and a maximum likelihood tree based on SNP alignment was constructed using RAxML-NG version 0.9.0 under the generalized time-reversible model with gamma-distributed rate heterogeneity (93). The phylogeny was tested against 100 bootstrap replications and the resulting tree was visualized with iTOL version 5.6.1 (94). The percent identity was automatically calculated from the core gene alignment by SNP-sites and a table generated from the Geneious software. Metadata of E. coli strains have been made freely publicly available for interactive exploration through Microreact (https://microreact.org/project/noM6Wi46mnpdYSzENnWmKX) (95). In addition to the general analysis of the strains (*n* = 167), the most represented pandemic clones (ST10, ST38, ST131, ST354, ST410, ST648, and ST744) were also analyzed separately.

### Data availability.

The data sets analyzed during the current study are available in the GenBank (https://www.ncbi.nlm.nih.gov/genbank) and corresponding access numbers are described in supplementary information (Table S1). Additionally, data are also available at the OneBR platform (http://onehealthbr.com).
