# Supplementary material for: WHO Critical Priority Escherichia coli as One Health Challenge for a Post-Pandemic Scenario: Genomic Surveillance and Analysis of Current Trends in Brazil
Source: Microbiol Spectr. 2022 Mar 2;10(2):e01256-21. doi: 10.1128/spectrum.01256-21 (PMC8941879; doi:10.1128/spectrum.01256-21)
Supplement: SUPPLEMENTAL FILE 1 — Supplemental material. Download SPECTRUM01256-21_Supp_1_seq11.pdf, PDF file, 0.4 MB [file spectrum01256-21_supp_1_seq11.pdf]

Supplemental Material

Supplementary Figure 1. Pangenome variation based on core and accessory genome components (gene presence/absence matrix), which was graphically represented using the roary_plots.py script. Panels show gene presence (dark blue) or absence (light blue) of the 167 *E. coli* strains.

Table S1. Epidemiological data and genomic repertoire (resistome, virulome, and plasmidome) of critical priority *Escherichia coli* across interconnected human-animal-food-environmental interfaces.

Table S2. Data obtained from genome-based phylogeny analysis of critical priority *Escherichia coli* population.

Table S3. Data obtained from genome-based phylogeny analysis of critical priority *Escherichia coli* population by predominant ST.

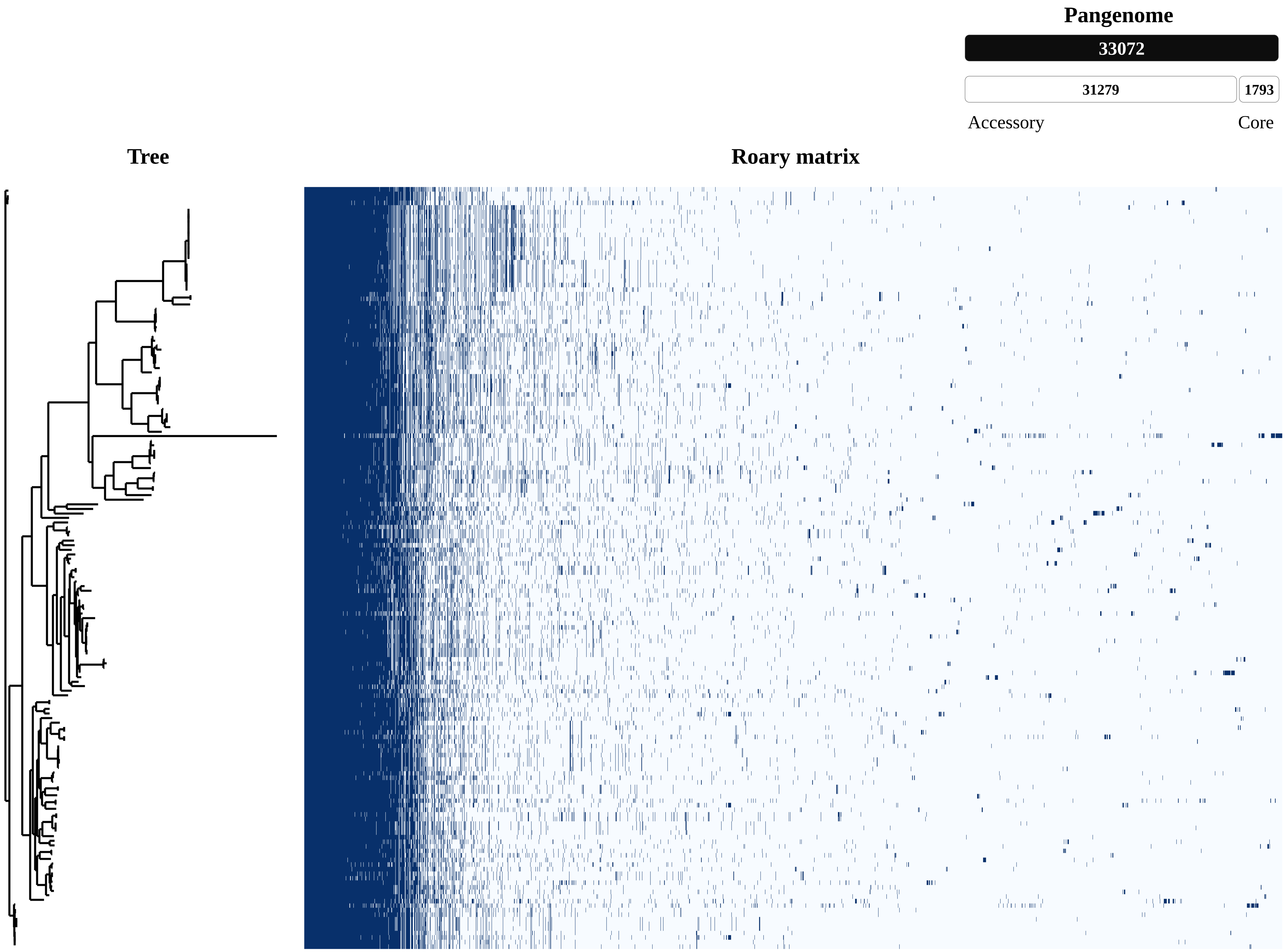

Supplementary Figure 1
